# Case report: Eczematous adverse drug reaction after selpercatinib treatment

**DOI:** 10.3389/fonc.2024.1475541

**Published:** 2024-12-24

**Authors:** Bingrun Li, Peng Cao, Wenjing Xu, Litao Zhang

**Affiliations:** ^1^ Graduate School, Tianjin University of Traditional Chinese Medicine, Tianjin, China; ^2^ Dermatological Department, Tianjin Academy of Traditional Chinese Medicine Affiliated Hospital, Tianjin, China

**Keywords:** eczematous reaction, adverse drug reaction, selective RET inhibitor, selpercatinib, glucocorticoid, case report

## Abstract

This study reports a 50-year-old patient presented with eczematous drug-eruption induced by selpercatinib after the treatment of non-small cell lung cancer (NSCLC). The patient has symmetric erythematous papules and plaques all over the body with dry, scaly skin accompanied by severe pruritus and visible scarring. After systemic treatment with glucocorticoids, the patient’ skin lesions were reduced well. Currently, the medical literature on the incidence of selpercatinib-induced cutaneous eczematous reactions and their clinical management is scarce. Therefore, this study provides novel evidence for the treatment of selpercatinib-induced cutaneous eczematous reactions.

## 1 Introduction

In 2012, the rearranged during transfection (RET) gene was first identified as an oncogenic driver in NSCLC (1). Mutations in the RET fusion gene have been observed in approximately 1% to 2% of NSCLC patients (1). Furthermore, at least 45 RET gene fusion partners have been identified in lung cancer, the most common of which is KIF5B-RET (70 - 90%) (2, 3). Selpercatinib (LOXO-292) is the first RET-specific kinase inhibitor approved by the FDA to treat RET-altered thyroid and NSCLC tumors (4). The LIBRETTO-001 trial indicated that selpercatinib has durable and safe anti-tumor activity as well as excellent intracranial efficacy (2, 5).

The LIBRETTO-001 trial also indicated that 44% of patients experienced treatment-emergent serious adverse events (SAEs), of which 11% were related to selpercatinib and drug hypersensitivity was the most common treatment-related SAE (2). Moreover, in clinical trials, 4.3% of selpercatinib-treated patients have indicated hypersensitivity of any grade, and the rash is among the most common symptoms (6). In addition, hypersensitivity reactions were relatively more severe when selpercatinib followed the treatment of immune checkpoint inhibitor (ICI) (7, 8). The literature has also indicated that the most common types of rashes induced by selpercatinib drug were erythematous, macular, maculopapular, morbilliform, and pruritic (6).

In this case, the patient underwent genetic testing, which revealed the presence of the KIF5B-exon24-RET-exon11 fusion variant (15.18% variant rate), and since the patient had already developed a secondary malignancy tumor in the brain, selpercatinib treatment was selected on a comprehensive basis. The patient had no history of ICI use and never developed a more severe generalized eczema-like flakiness and dry skin. This is the first report of such cutaneous toxicities associated with selpercatinib treatment.

## 2 Case report

### 2.1 Case description

A 50-year-old patient was diagnosed with a secondary malignant tumor of the frontal lobe and NSCLC via genetic testing. The patient was prescribed 4 weeks of selpercatinib as a subsequent treatment. However, after 4 weeks, the patient indicated the development of eczematous skin lesions.

**Figure 1:**
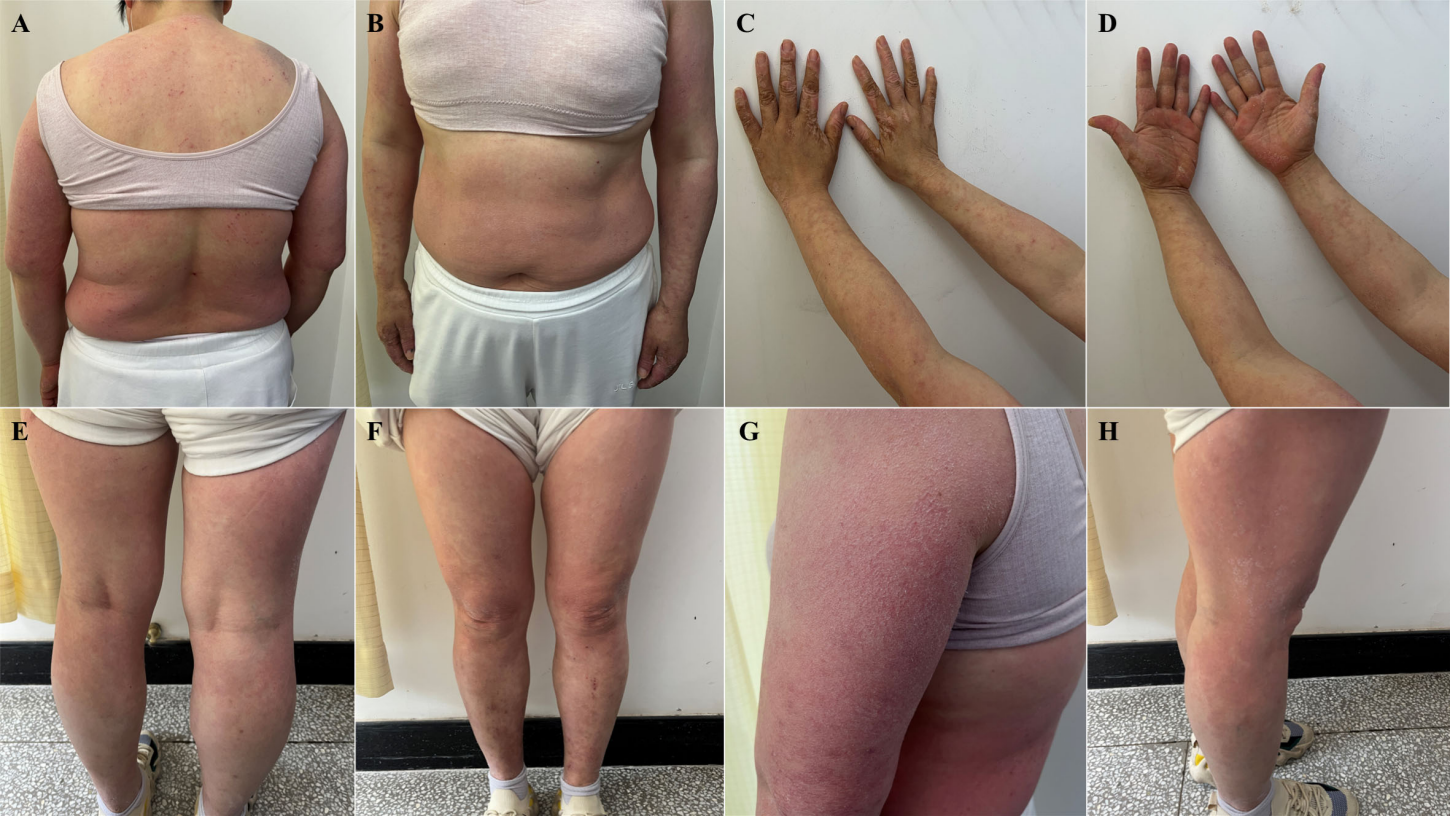
Eczematous skin lesion: symmetrical erythematous papules and plaques throughout the body, with dry and scaly skin, as well as severe pruritus and visible scratch marks (May 29, 2024). **(A)** torso front, **(B)** back of torso, **(C)** forearm flexion, **(D)** forearm extension, **(E)** lower extremity frontal, **(F)** lower extremity dorsal, **(G)** lateral aspect of the left upper arm with the most typical lesions, **(H)** lateral right thigh with more severe lesions.

The patient started taking selpercatinib 160 mg, PO, BID on April 17, 2024 and developed erythematous papules on the face and neck, with intolerable itching around May 15, 2024. Routine blood tests revealed that the patient had no abnormalities. The patient self-administered oral prednisone acetate tablets, 5 mg irregularly for over a week, which resolved skin lesions on the face and neck. However, eczema-like changes subsequently manifested on the abdomen, back, and extremities, with the most severe skin lesions observed on the hands, exhibiting significant cracking and desquamation. For proper treatment, the patient was admitted to Tianjin Academy of traditional Chinese Medicine Affiliated Hospital on May 29, 2024. The dermatological examination revealed symmetrical erythematous papules and plaques throughout the body, with dry and scaly skin, as well as severe pruritus and visible scratch marks (**Figure 1**).

### 2.2 Laboratory analyses

Dermatopathologic biopsy specimens were taken from the leg. The microscopic analysis of PAS stained (-) tissues revealed the presence of epidermal hyperkeratosis, hyperkeratosis, superficial crusting, hypertrophy of the stratum spinosum, and mass infiltration of large numbers of lymphocytes, histiocytes, and small numbers of plasma cells around the superficial dermal vessels. Furthermore, laboratory tests revealed mild eosinophilia [0.57 * 10^9^/L (normal, 0.02-0.52)], 1.2% basophils (normal, 0-1), and 117.20 KIU/L serum IgE (normal, 0-100). Clinical, histologic, and laboratory findings were consistent with a diagnosis of eczema.

### 2.3 Treatment regimen

Considering the patient’s physical condition, the treatment regimen included: compound betamethasone intramuscular injection (1 mL of 5 mg betamethasone dipropionate with 2 mg betamethasone sodium phosphate, both measured as betamethasone); ST, topical application of topical 1:1 mixture of triamceinolone acetonide acetate cream (1.5%) and allantoin cream; BID. In the case of intense itching at night, which interferes with normal sleep, additional chlorphenamine maleate tablets 5 mg were prescribed; PO, QN.

### 2.4 Follow-up

**Figure 2:**
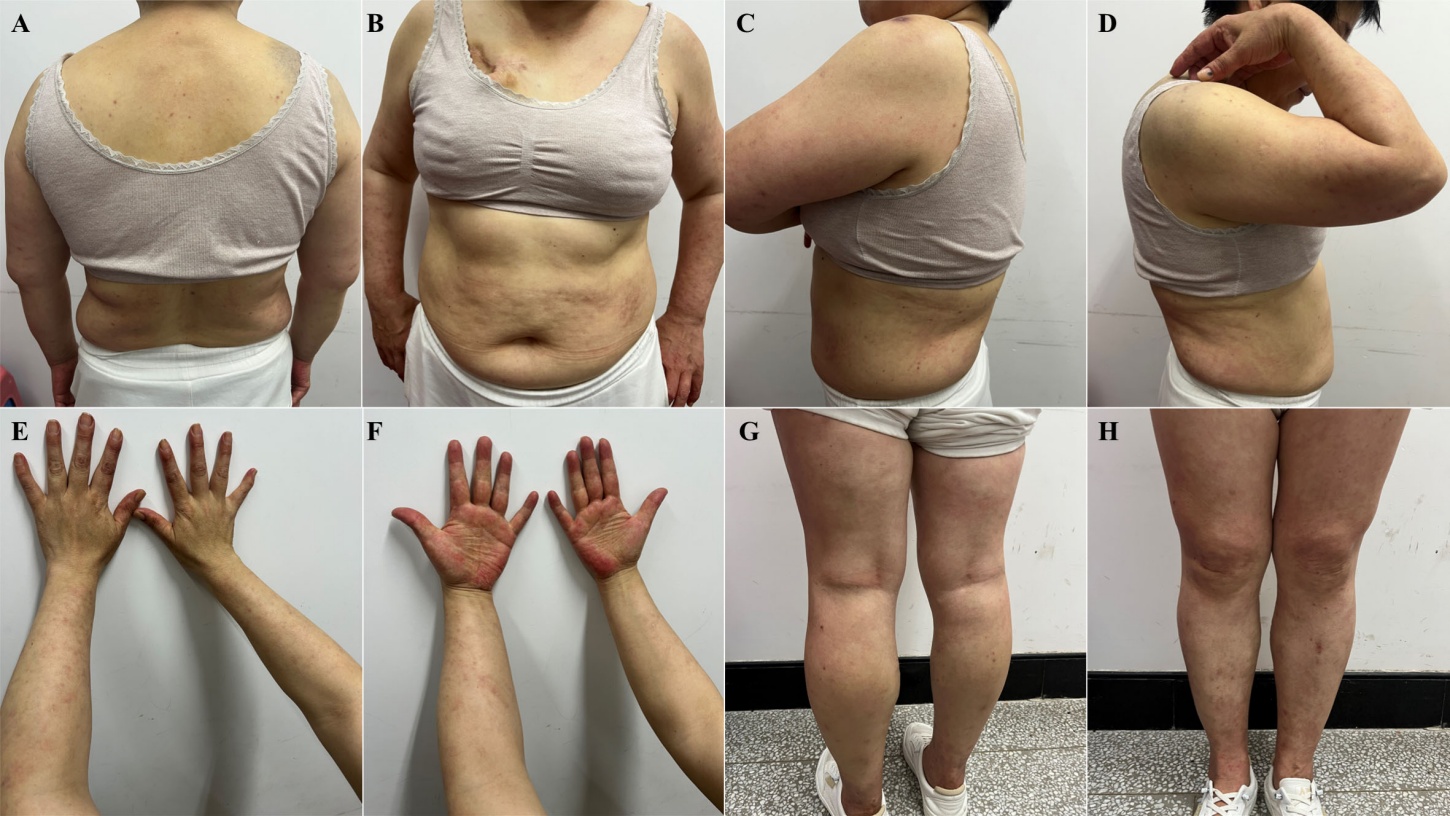
Significant improvement of skin lesions after corticosteroid drug treatment (June 7, 2024). **(A)** torso front, **(B)** back of torso, **(C)** left lateral upper arm, **(D)** right lateral upper arm, **(E)** forearm flexion, **(F)** forearm extension, **(G)** lower extremity frontal, **(H)** lower extremity dorsal.

After 10 days, on June 7, 2024, the patient returned to the clinic with significantly improved skin lesions and approaching normal skin morphology (**Figure 2**). Patients did not discontinue or taper selpercatinib throughout treatment.

## 3 Discussion

Skin damage is a common adverse effect of oncology therapeutic agents, especially ICI and multi-kinase inhibitors, and eczematous damage has also been reported (9, 10). The anti-tumor mechanism of ICI involves the co-stimulation of T-cells to increase immune activation, which promotes anti-tumor immune responses (11). ICI promotes widespread and relatively nonspecific activation of T cells in patients, inducing immune system hyperfunction, which consequently leads to the development of immune-related adverse events such as eczema (11). Multi-kinase inhibitors achieve anti-tumor effects by inhibiting the epidermal growth factor receptor (EGFR) signaling pathway (6). EGFR is crucial for epidermal regeneration and skin physiology maintenance. Furthermore, its signaling dysregulation is significantly associated with the pathogenesis of inflammatory skin diseases (12). A report from the United States described a severe systemic hypersensitivity reaction following the use of selpercatinib in a patient with skin lesions similar to those associated with drug-induced hypersensitivity syndrome (13). Furthermore, the report indicates a correlation between prior ICI therapy and the incidence of hypersensitivity adverse drug reactions. In light of this, we postulate that although selpercatinib is a highly selective RET inhibitor with minimal effect on other kinases, and has a lower risk of skin-related adverse events compared to ICIs and multikinase inhibitors, they cause similar manifestations of skin damage and are all associated with prior ICI exposure (8). Consequently, there may be a similar pathogenic mechanism.

Over the past few years, several studies have reported that targeted therapies against psoriasis cause eczematous damage, specifically the anti-tumor necrosis factor alpha (TNF-α) and anti-interleukin 17 (IL-17) therapies with ustekinumab, secukinumab, ixekizumab, etc. targeted therapeutic agents (14, 15). Psoriasis and eczema are thought to be diseases caused by an imbalance in the T helper (Th)1/Th2 immune response, with Th1 being more prominent in psoriasis and Th2 predominating in eczema (16). The Th1 and Th2 pathways are closely related and are in homeostasis, where, when one pathway is blocked or reduced, the response of the other pathway is more euphoric (17). Therefore, psoriasis monoclonal antibody therapy, whether directed against TNF-alpha or IL-17, is directed against the inhibition of the Th1 pathway and will increase the response of the Th2 pathway, which results in the development of Th2-type disease, such as eczematous damage (16). Selpercatinib, prescribed in this case report, is also a targeted drug. However, unlike the aforementioned monoclonal antibodies, selpercatinib is a small-molecule kinase inhibitor with high selectivity against RET gene fusions and directly targets the Th1 pathway. It has been observed that IL-22 levels are elevated in eczematous lesions induced by anti-TNF-α therapy. IL-22 in eczematous lesions has been observed to be associated with drug-induced adverse reactions (16). Therefore, it can be hypothesized that selpercatinib also affects IL-22 or other functionally similar cytokines that result in the development of eczematous skin damage.

In this case report, the role of estrogen in the occurrence of adverse drug events was also studied. Estrogen plays a significant role in the pathogenesis and prognosis of eczema and other dermatological conditions by facilitating the formation and restoration of the skin barrier by regulating filamentous polyprotein expression and stimulating β-glucosidase (18). The association of RET with estrogen receptors (ER) has been substantiated, with both exhibiting co-expression, and RET is considered an essential factor in the development of ER-positive breast cancer (19). In addition, estrogen directly modulates the effects on RET expression (20). Therefore, RET inhibition induced by selpercatinib may directly or indirectly affect estrogen levels by altering ER expression, which in turn destabilizes the skin barrier, thereby promoting eczematous damage. Although there are clinical reports indicating the efficacy of selpercatinib in the treatment of RET fusion-positive breast cancer (21), the number of articles is limited, and the effects of this RET inhibitor on patient estrogen levels remain under-investigated. Further research is required to confirm the effect of selpercatinib on estrogen levels and to elucidate the mechanism of adverse skin reactions. This will necessitate the collection of extensive genomic data and clinical data.

## 4 Conclusion

Subsequently, the patient used only topical glucocorticoid cream, with a gradual reduction in concentration and dosage. The follow-up to date has indicated that the rash is well controlled, there are no other complaints of discomfort, and selpercatinib administration was not disturbed. Furthermore, an additional report has documented the efficacy of glucocorticoid therapy for the treatment of cutaneous adverse reactions caused by the use of selpercatinib (13). This may indicate the feasibility of using glucocorticoids to treat selpercatinib-induced adverse drug reactions in the cutaneous region.

Reports of eczematous adverse drug reactions following the use of selpercatinib are uncommon. This paper presents a speculative exploration of the mechanism of eczematous cutaneous adverse reactions following selpercatinib treatment. Additionally, it documents the protocol of clinical topical glucocorticoid management of this adverse reaction, with the aim of informing oncologists and dermatologists. Individual reports are inevitably limited, further research on the mechanism of eczematous eruption is required to manage this adverse drug reaction better and alleviate the patient’s skin discomfort while avoiding interruptions or dose reductions of the primary regimen.

## Data Availability

The original contributions presented in the study are included in the article/supplementary material. Further inquiries can be directed to the corresponding author.
